# Successful treatment of cardiac arrest following hysteroscopic surgery using extracorporeal membrane oxygenation: A case report

**DOI:** 10.1097/MD.0000000000025519

**Published:** 2021-04-16

**Authors:** Ting Chen, Li Yao, Fei Tong, Chunyan Zhu

**Affiliations:** a Intensive Care Unit, The Second People's Hospital of Hefei; b Intensive Care Unit, The First Affiliated Hospital of USTC, University of Science and Technology of China, Anhui, China.

**Keywords:** hyponatremia, hysteroscopic surgery, veno-venous ECMO, water intoxication syndrome

## Abstract

**Rationale:**

Cardiac arrest caused by water intoxication syndrome following hysteroscopic surgery is a rare but life-threatening occurrence. Extracorporeal membrane oxygenation (ECMO) is rarely used to treat water intoxication syndrome in hysteroscopic surgery. Here, we successfully treated a patient with water intoxication syndrome following hysteroscopic surgery with ECMO.

**Patient concerns:**

We report a rare case of cardiac arrest during hysteroscopic surgery treated with veno-venous (VV) ECMO.

**Diagnosis:**

Water poisoning syndrome was diagnosed by electrolyte examination, the lowest value of serum sodium was 110.7 mmol/L.

**Interventions:**

VV-ECMO was prescribed as a measure after traditional cardiopulmonary resuscitation.

**Results:**

ECMO was successfully evacuated on day 5 and the patient was discharged on day 45.

**Conclusion:**

Mastering the hysteroscopic operative techniques and using a bipolar hysteroscopic generator, isotonic fluid, perfusion pressures less than 100 mm Hg, and local anesthesia may reduce the risk of hysteroscopic water intoxication syndrome. During hysteroscopic surgery, patients may experience cardiac arrest and fatal water intoxication syndrome. Even when traditional cardiopulmonary resuscitation is successful, VV ECMO may contribute to the recovery of brain function if oxygenation is not maintained.

## 1 Introduction

The hysteroscope has been used in obstetrics and gynecology departments for decades and is one of the most commonly used surgical instruments. Because of the small amount of necessary trauma and rapid patient recovery, hysteroscopic surgery is often used in gynecological and obstetric pathological diagnosis and treatment, such as in myomectomy, congenital mediastinal uterine correction, sterilization, and endometrial ablation.^[1]^ However, complications, such as uterine perforation, bleeding, dilute hyponatremia, gas embolism, cardiac arrest, and even death, are not uncommon.^[2]^ In the last 20 years, extracorporeal membrane oxygenation (ECMO) has become widely used in refractory reversible cardiopulmonary diseases, such as acute respiratory distress syndrome, massive pulmonary embolism, and fulminant myocarditis.^[3]^ However, ECMO is rarely used in the treatment of patients who experience cardiac arrest during hysteroscopic surgery. We report a case of successful use of veno-venous (VV) ECMO to rescue and treat a patient with water intoxication syndrome.

## 2 Case report

**Figure 1:**
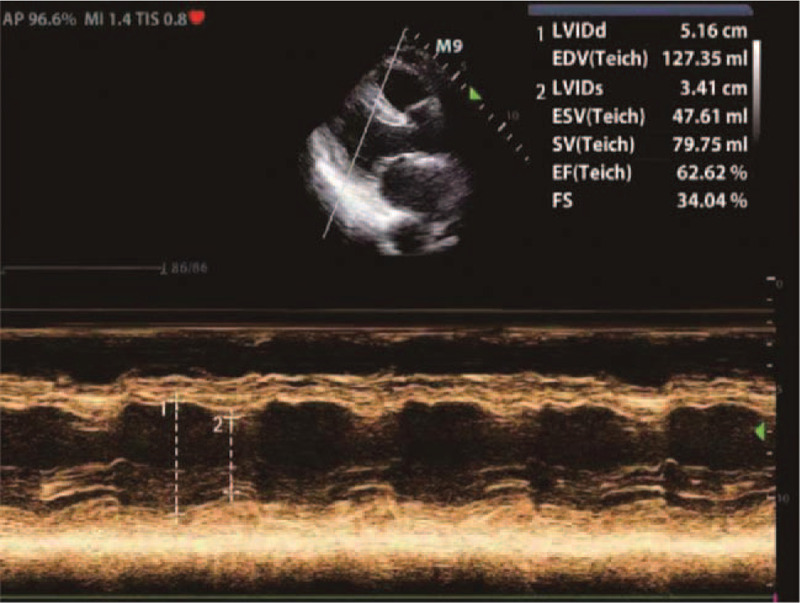
Echocardiography showing a left ventricular ejection fraction (LVEF) of 62.62%.

**Figure 2:**
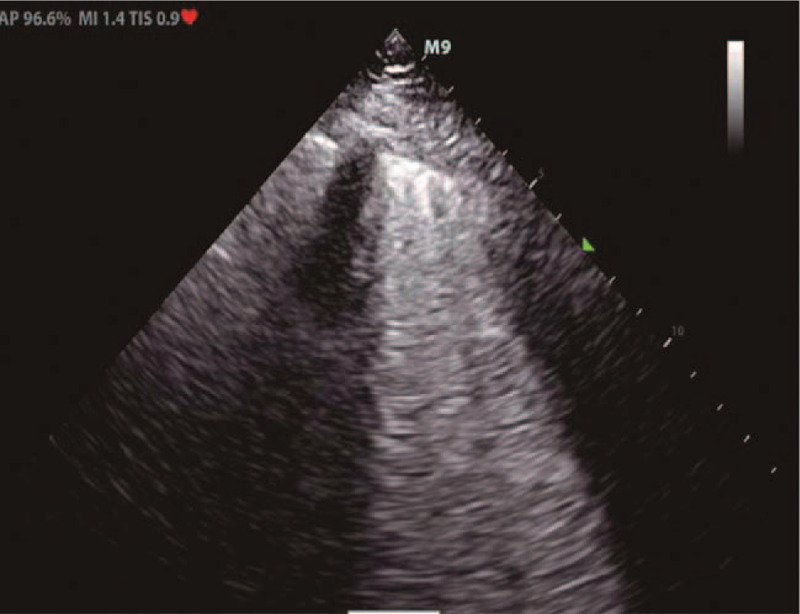
Lung ultrasonography showing a large number of B lines.

**Figure 3:**
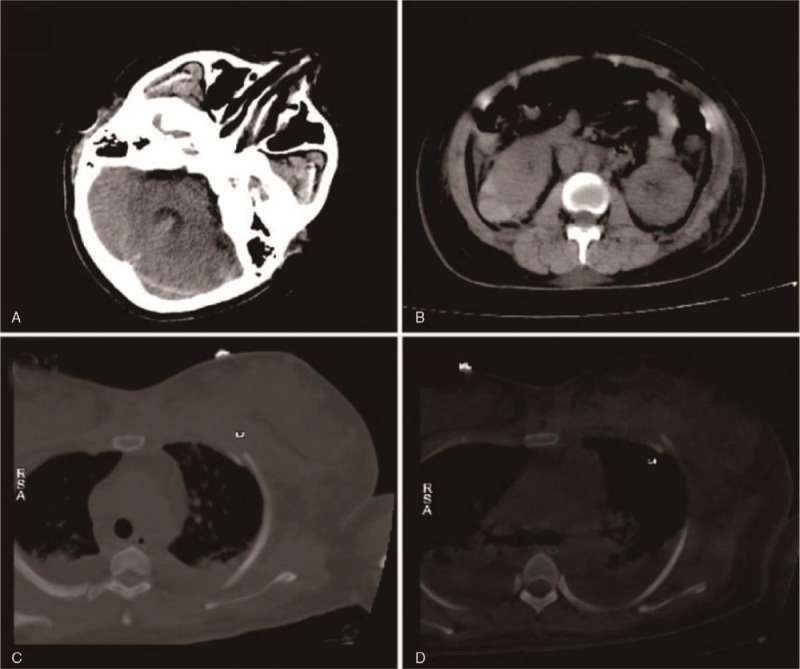
Computed tomography showing subarachnoid hemorrhage and right renal capsule hematoma.

A 36-year-old woman with height of 160 cm and weight of 62 kg was admitted to the hospital because of a 4-year history of uterine fibroids and 1-month history of frequent urination. Multiple uterine fibroids and incomplete uterine mediastinum were found on ultrasound examination. Routine blood examination findings, liver and kidney function, electrolyte levels, and coagulation function were in the normal range preoperatively. On the third day of admission, hysteroscopic mediastinotomy combined with myomectomy was performed under general anesthesia. During the operation, 7500 mL 1.5% glycine infusion solution was used, the uterine distention pressure was 100 mm Hg, and the difference in uterine distention liquid was 1500 mL. After the operation, the patient experienced sudden cardiac arrest; cardiopulmonary resuscitation (CPR) comprising chest cardiac compression, epinephrine, furosemide, and 25% mannitol, was applied immediately; 200 J electric defibrillation was performed 3 times. Blood gas analysis revealed the following: pH, 7.026; Na^+^, 110.7 mmol/L; K^+^, 4.90 mmol/L; base excess, −11 mmol/L; and lactic acid, 18 mmol/L. Twenty-six minutes after CPR was commenced, the patient's spontaneous heartbeat recovered. Electrocardiogram monitoring revealed a heart rate of 100 beats/min, the blood pressure was 95/58 mm Hg (epinephrine 0.4 μg/kg/min, norepinephrine 1.0 μg/kg/min), and the peripheral oxygen saturation was 73%. Physical examination found a bilateral pupil diameter of 5 mm and lack of a direct light reflex, a large number of blister sounds on double lung auscultation, pink foamy liquid in the tracheal cannula, terminal cyanosis of the lips and extremities, facial swelling, and a Glasgow coma score of E1VTM1. Blood gas analysis after initial resuscitation revealed pH 7.296, PO_2_ 42.7 mm Hg, PCO_2_ 51.1 mm Hg, base excess −15 mmol/L, and Na^+^ 118.1 mmol/L. The left ventricular ejection fraction was 62.62% on echocardiography (Fig. 1). Furosemide 200 mg, 5% bicarbonate injection 375 mL, and 3% sodium chloride injection 100 mL were prescribed. The parameters of the ventilator were adjusted to an oxygen concentration of 100%, respiratory rate of 20 beats/min, positive end-expiratory pressure of 10 cmH_2_O, and an inspiratory time of 1.0 second. Oxygenation did not improve and pulmonary ultrasound revealed a significant number of B lines (Fig. 2). The indication for acute VV ECMO was confirmed by the ECMO team. We installed the VV ECMO equipment (centrifugal pump, Maquet Rotaflow RF 32; Maquet Cardiopulmonary AG, Hirrlingen, Germany) at the bedside. A 17-French ECMO catheter (Duraflo, Edwards Lifesciences; Irvine, CA) was inserted via the right internal jugular vein, between the superior vena cava and right atrium; a 19-French catheter was inserted into the femoral vein and advanced under B-mode ultrasound guidance. Both catheters were then connected to their respective ECMO sheaths. The ECMO was set to a flow rate of 4.0 L/min, with a pump speed of 3000 r/min, return pressure of 120 mm Hg, and access pressure of −40 mm Hg. No anticoagulation was selected within 24 hours because of the bloody fluid in the catheter and heparin-coated lung. After 4 hours of ECMO, the blood pressure was maintained at 100/65 mm Hg. Epinephrine was gradually stopped, and norepinephrine was gradually reduced to 0.4 μg/kg/min. The patient was transferred to the intensive care unit (ICU). Subsequent examination revealed radial artery blood gas pH, 7.30; PO_2_, 62 mm Hg; PCO_2_, 32 mm Hg; lactic acid, 15 mmol/L; base excess, −8.5 mmol/L; Na^+^, 130 mmol/L; creatinine, 235 μmol/L (normal value <80 μmol/L); free hemoglobin, 84 mg/L (normal value; 0–40 mg/L); prothrombin time, 23 seconds; and activated partial thromboplastin time (APTT), 120 seconds. The oxygen concentration of the ventilator was adjusted to 40%, lung recruitment strategy, and head hypothermia brain protection, blood transfusion, infection prevention, diuresis, and maintaining the stability of the internal environment were treated. After 12 hours of ECMO, the patient could open their eyes in response to calls and partially obeyed the doctor's commands. After 24 hours of ECMO, heparin was used for anticoagulation, and the APTT was maintained at 55 to 60 seconds. Concomitantly, norepinephrine was gradually reduced and stopped, serum sodium (137 μmol/L) returned to the normal range. On the 5th day of VV ECMO, a small amount of exudation was seen on chest X-ray, and the heart rate, blood pressure, and peripheral oxygen saturation did not change after 2 hours of oxygen deprivation; blood gas analysis revealed pH, 7.36; PO_2_, 86 mm Hg; and PCO_2_, 38.2 mm Hg. The patient was weaned from VV ECMO safely. Thrombi in the right internal jugular vein and the left lower limb intermuscular vein were detected using bedside ultrasonography. Low molecular weight heparin was used to anticoagulate for (4000 U every 12 hours). On the 7th day of admission to the ICU, the patient was weaned from the ventilator after a spontaneous breathing test. At the same time, computed tomography (CT) revealed a small subarachnoid hemorrhage, a small hematoma in the right renal capsule, and multiple rib fractures (Fig. 3); the anticoagulant drug was changed to rivaroxaban 20 mg once per day. After 18 days in the ICU, the patient was transferred back to the Obstetrics and Gynecology Department for further treatment. One month later, ultrasound examination revealed that the lower extremity venous thrombosis had disappeared, the right internal jugular vein was partially recanalized, and the head CT revealed no abnormality. After 45 days, the patient was discharged and followed up for 6 months.

## 3 Discussion

Hysteroscopic surgery has been recognized as the standard approach for the diagnosis and treatment of various gynecological diseases in gynecology and obstetrics. It has been widely used for conditions ranging from early dysfunctional uterine bleeding to the treatment of uterine fibroids and adenoids. At present, significant practical experience has been accumulated. Gynecologists should perform at least 250 hysteroscopic examinations before performing hysteroscopic surgery and use different kinds of dilating media and different electrodes to reduce the complications of surgery.^[4,5]^ The overall incidence of complications is 0.28% to 10%, and includes bleeding, uterine perforation, hyponatremia, air embolism, and even death.^[2,5,6]^ A previous report^[7]^ stated that all patients with cardiac arrest were treated using venoarterial ECMO, that is, extracorporeal cardiopulmonary resuscitation (ECPR), the use of VV ECMO to treat cardiac arrest water by intoxication syndrome caused has not been reported during hysteroscopic surgery has not been reported. With the help of a multidisciplinary team, we successfully used VV ECMO to treat a case of water intoxication syndrome during hysteroscopy.

Water intoxication syndrome is a serious complication of hysteroscopic surgery. The pathogenesis is similar to that of transurethral resection syndrome seen after transurethral resection of prostate, with an incidence rate of 0.02% to 0.2%.^[8]^ Many factors lead to the development of water intoxication syndrome.^[9]^ Among them, the uterine distention medium, which can be divided into high viscosity and low viscosity media, is a particularly important factor. High viscosity media include low molecular dextran, which can easily cause coagulation disorders and liquid overload, and are not commonly used in the clinic. Low viscosity media include 1.5% glycine, 3% sorbitol, and 5% mannitol, among other, and are the first choice for hysteroscopic surgery in gynecology. However, they can lead to potentially fatal fluid overload and diluted hyponatremia because of the lack of electrolytes; 0.9% normal saline is commonly used as an isotonic electrolyte liquid for uterine distention and will only volume overload and reduce the perioperative risk; however, a bipolar hysteroscopic electric generator is required to complete the operation.^[10]^ In this case, we selected 1.5% glycine as the dilating media and used a monopole hysteroscopic generator.

After the operation, cardiac arrest occurred, and auxiliary examination revealed severe hyponatremia, metabolic acidosis, hemolysis, and coagulation dysfunction. At the same time, prior studies have shown that the occurrence of water intoxication syndrome is related to the intrauterine perfusion pressure. The uterine perfusion pressure in the patient is 100 mm Hg, and is generally maintained at 80 to 150 mm Hg^[9]^; however, a uterine perfusion pressure of 60 to 75 mm Hg can be sufficient to complete most surgical operations. Reducing the pressure may help reduce the occurrence of water intoxication syndrome.

Operation time is also an important factor related to its occurrence. An operation time of more than 60 minutes is a high-risk factor for water intoxication syndrome.^[11]^ The operation time, in this case, was 45 minutes, which indicated a moderate risk of water intoxication syndrome. Bergeron et al^[12]^ compared the relationship between local anesthesia and general anesthesia with the occurrence of water intoxication syndrome in hysteroscopic surgery. It was found that the incidence of water intoxication syndrome with local anesthesia was lower than that with general anesthesia. In this case, our choice of general anesthesia may have increased the risk of occurrence of water intoxication syndrome.

ECMO comprises the extracorporeal circulation equipment with a lung membrane and centrifugal pump as the core components and can save time when treating reversible heart and lung diseases.^[3]^ With progressive developments in ECMO, this approach has been used for a wider range of purposes from initial cardiopulmonary function support after major cardiac surgery and in patients with end-stage structural lung disease waiting for lung transplantation to treating various critical diseases, such as acute myocardial infarction, severe fulminant myocarditis, and poisoning, and after CPR.

ECMO is playing an increasingly pivotal role in clinical practice and is often used in CPR. Venoarterial ECMO, that is, ECPR, is defined as ECMO hemodynamic support during CPR before hemodynamic recovery.^[7]^ The use of VV ECMO has been less frequently reported after successful CPR. After the success of traditional CPR, the lowest blood sodium level was 110.7 mmol/L in the blood gas analysis. After diuresis, infusion of 3% sodium chloride and glucocorticoids, and adjustment of ventilator parameters, the oxygenation index was less than 100. Kollengode et al^[13]^ summarized 280 cases of pregnant women receiving ECMO treatment from 1997 to 2017; among them, the indications for VV-ECMO including:

1. Severe respiratory failure (but may be reversible);
2. Hypercapnia with severe respiratory acidosis, although the optimal conventional mechanical ventilation and breathing frequency are greater than 35 times/min;
3. Despite best invasive ventilation support (such as low tidal volume, appropriate positive end-expiratory pressure, lung recruitment strategy, prone position ventilation, etc), oxygen concentration ≥0.9, peep ≥10 cmH_2_O, but PaO_2_/FiO_2_ (oxygenation index) <100.

This case is consistent with criteria 1 and 3; before ECMO, the left and right ventricular contractions were normal, the left ventricular ejection fraction was 62.5%, and stroke volume was 62 mL. Therefore, we chose VV ECMO as an assistive therapy. Chen et al^[14]^ confirmed that patients treated with ECPR had better short-term and long-term survival than those treated with traditional CPR. However, VV ECMO may reduce brain damage after resuscitation in some patients, such as those with gas embolism, decompression sickness, and severe diluted hyponatremia. Nonetheless, this requires further research.

ECMO-related complications, including hemorrhage, thrombosis, and thrombocytopenia, are not uncommon. In this case, all the above complications occurred. Bleeding occurred at the right femoral vein of the puncture catheter, and the hematoma did not increase significantly after local compression. The puncture site injury was thought to be related to the postoperative coagulation dysfunction. After the patient's coagulation function had significantly improved, ECMO was anticoagulated with unfractionated heparin for 48 hours, maintaining an APTT of 55 to 60 seconds, which met the requirements for ECMO anticoagulation management guidelines provided by the Extracorporeal Life Support Organization^[15]^; the use unfractionated heparin was beneficial for clinical monitoring and provision of neutralizing drugs. After ECMO was removed, the routine CT scan revealed a small subarachnoid hemorrhage and small hematoma in the right renal capsule, which may have been related to CPR and ECMO.

Thrombosis is also a common complication of ECMO. It mainly manifests as the formation of a thrombus at the puncture site, which may lead to pulmonary embolism, and is potentially life-threatening. Before withdrawing ECMO, we routinely performed vascular ultrasound examinations and found a thrombus in the right internal jugular vein and left lower limb intermuscular vein. We treated the patient with low molecular weight heparin and then changed the treatment to argatroban anticoagulation. Thirty days later, the left lower limb intramuscular vein thrombosis had disappeared, and the right internal jugular vein was recanalized.

Thrombocytopenia is also a common complication of ECMO. Thrombocytopenia is mainly ECMO centrifugal pump, pipeline adsorption, and activated coagulation. If the platelet count is less than 20,000 /mL, platelet transfusion is required. While our patient was thrombocytopenic, her platelets were maintained at ≥40,000/mL), and transfusion was unnecessary

In conclusion, the incidence of severe water intoxication syndrome and cardiac arrest in hysteroscopic surgery is low. However, when it occurs, it seriously endangers the health of patients and may even cause death. During hysteroscopic surgery, mastering the hysteroscopic operative techniques and using a bipolar hysteroscopic generator, isotonic fluid, perfusion pressures less than 100 mm Hg, and local anesthesia may reduce the risk of hysteroscopic water intoxication syndrome. During hysteroscopic surgery, patients may experience cardiac arrest and fatal water intoxication syndrome. Even when traditional CPR is successful, VV ECMO may contribute to the recovery of brain function if oxygenation is not maintained.

## Acknowledgments

We thank Kelly Zammit, BVSc, from Liwen Bianji, Edanz Editing China (www.liwenbianji.cn/ac), for editing the English text of a draft of this manuscript.

## Author contributions

**Conceptualization:** Ting Chen, Fei Tong.

**Data curation:** Chunyan Zhu, Ting Chen, Li Yao, Fei Tong.

**Formal analysis:** Ting Chen, Li Yao.

**Investigation:** Ting Chen.

**Methodology:** Ting Chen.

**Resources:** Chunyan Zhu, Li Yao, Fei Tong.

**Supervision:** Chunyan Zhu, Fei Tong.

**Validation:** Chunyan Zhu, Ting Chen, Fei Tong.

**Visualization:** Li Yao, Fei Tong.

**Writing – original draft:** Chunyan Zhu, Ting Chen, Li Yao, Fei Tong.

**Writing – review & editing:** Chunyan Zhu, Ting Chen.
